# Effectiveness of the Modification of Sewers to Reduce the Reproduction of *Culex pipiens* and *Aedes albopictus* in Barcelona, Spain

**DOI:** 10.3390/pathogens11040423

**Published:** 2022-03-31

**Authors:** Tomas Montalvo, Agustin Higueros, Andrea Valsecchi, Elisenda Realp, Cristina Vila, Alejandro Ortiz, Víctor Peracho, Jordi Figuerola

**Affiliations:** 1 Agència de Salut Publica de Barcelona, Plaça Lesseps 1, 08023 Barcelona, Spain; tmontal@aspb.cat (T.M.); avalsecc@aspb.cat (A.V.); erealp@aspb.cat (E.R.); vperacho@aspb.cat (V.P.); 2 CIBER Epidemiología y Salud Pública (CIBERESP), Calle Monforte de Lemos 5, 28029 Madrid, Spain; 3 Barcelona Cicle de l’Aigua, Ajuntament de Barcelona, Carrer de l’Acer 16, 08038 Barcelona, Spain; ahigeroc@bcn.cat (A.H.); cvilaru@bcn.cat (C.V.); aortizg@bcn.cat (A.O.); 4 Estación Biológica de Doñana, Avenida Américo Vespucio 26, 41092 Sevilla, Spain

**Keywords:** mosquito control, *Aedes albopictus*, *Culex pipiens*, integrated mosquito management, manipulation of mosquito habitat, invasive *Aedes*

## Abstract

Mosquitoes breeding in urban sewage infrastructure are both a source of nuisance to the local population and a public health risk, given that biting mosquitoes can transmit pathogenic organisms to humans. The increasing presence of the invasive mosquito species *Aedes albopictus* in European cities has further exacerbated the problems already caused by native *Culex pipiens*. We tested the effectiveness of modifications to sewage structures as an alternative to the use of biocides to prevent mosquito breeding. The placing of a layer of concrete at the bottom of sand sewers to prevent water accumulation completely eliminated mosquito reproduction, and so eliminates the need for biocides in modified structures. Sewer modification is thus a valid low-cost alternative for mosquito control.

## 1. Introduction

Mosquitoes are an important source of nuisance to human populations due to the biting behaviour of their females and their involvement in the transmission of pathogens, which are responsible for more than 700,000 human deaths/year [1]. While the density of mosquitoes in urban areas tends to be lower than in rural areas [2], this is not always true, and some species have found suitable areas for reproduction close to human dwellings. This is the case of *Culex pipiens* (Linnaeus, 1758), a widely distributed mosquito that is competent for the transmission of at least 20 viruses that are relevant to public and animal health [3]. Some invasive species of the genus *Aedes* have also adapted particularly well to breeding in urban areas due to their capacity to use a variety of small water bodies to lay their eggs [4]. This has facilitated the establishment of invasive populations in several continents by species such as *Aedes aegypti* (Linnaeus, 1762) and *Ae. albopictus* (Skuse, 1894) [5]. Due to their capacity to transmit agents causing serious diseases, the establishment of these species in Europe and America has led to worrying outbreaks of diseases such as Dengue, Chikungunya and Yellow Fever [6,7]. For these reasons, major efforts are being put into the control of their populations [8]. A recent analysis estimated that between 1970 and 2017 the damage caused by and the management of invasive mosquitoes belonging to the genus *Aedes* had an accumulated cost of approximately USD 150 billion [9]. In Spain, an outbreak of West Nile virus in 2020 produced 77 recorded human cases of severe disease, including eight deaths due to the transmission of this virus by native mosquitoes of the genus *Culex* [10]. In addition, the autochthonous transmission of Dengue was recorded in 2018, originating from imported cases in areas with *Ae. albopictus* activity [11]. Reducing both native and invasive mosquito populations in urban areas is key to reducing the risk of pathogen transmission and nuisance to citizens. According to Spanish law, mosquito control is the responsibility of local authorities in public areas and of individual citizens in private areas (i.e., Ley de Salud Pública de Catalunya 18/2009, 28 October 2009). In public areas, sewers provide mosquitoes with suitable places in which to breed, and numerous towns and cities run surveillance and control programs that include, among other measures, the regular treatment of sewers with biocides at considerable economic expense [12]. *Ae. albopictus* was detected for the first time in Catalonia in 2004 [13] and in the city of Barcelona in 2005 [14]. In addition to *Ae. albopictus*, *Cx. pipiens* and *Culiseta longiareolata* (Macquart, 1838) are frequently recorded breeding in public places [15]. Local authorities receive over 200 complaints/year regarding the proliferation of mosquitoes [16]. Due to the spread and expansion of *Ae. albopictus*, a control program was established in 2004 in Barcelona that consists of the surveillance and control of mosquitoes in the city’s streets and sewage system. It includes a prevention strategy aimed at reducing the impact of these vectors on the human population, and the application of biocides at potential breeding sites (more than 12,000 sewers and 300 ornamental fountains) to avoid the proliferation of mosquitoes. The control of breeding sites is linked to a program for monitoring and controlling mosquito-borne arboviruses, with special attention being paid to those that could be transmitted by *Ae. albopictus,* to restrict the risk of autochthonous transmission of imported mosquito-borne diseases into the city [16].

In this paper, we describe the results of a pilot project in the city of Barcelona (Spain) designed to cut the costs of mosquito control and reduce mosquito reproduction in cities by modifying the structures of sewers. Specifically, we hypothesize that a small modification in sewer structures may prevent mosquitoes from breeding there.

## 2. Results

**Figure 1:**
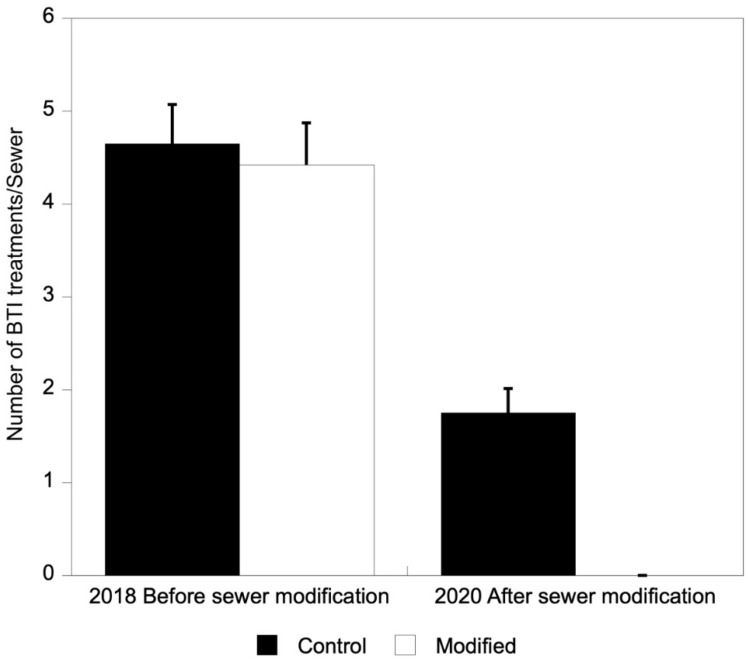
Mean number ± s.e. of BTI treatments applied in control and modified sewers in 2018 (before sewer modification) and in 2020 (after sewer modification).

The higher rainfall recorded in 2018 than in 2020 resulted in a greater frequency of water present in sand sewers in 2018 than in 2020; as well, there was a greater frequency of larvae and, consequently, treatments were applied more often (Table 1). A significant interaction between year and sewer modification was found for water presence and the frequency of *Bacillus thuringiensis israelensis* (BTI) applications (Figure 1), as well as for larvae presence (Table 1). These interactions occurred because in 2018, previous to modification, no differences were found between control and modified sewers in terms of sewer flooding (F_1,73.79_ = 0.58, *p* = 0.45), treatment frequency (F_1,73.70_ = 0.13, *p* = 0.72) or larval presence (F_1,72.52_ = 0.60, *p* = 0.44). However, in 2020 the control sewers were more frequently flooded (F_1,73.79_ = 44.77, *p* < 0.0001), more frequently treated (F_1,73.70_ = 13.64, *p* = 0.0004) and had more larvae (F_1,72.52_ = 3.87, *p* = 0.05) than the modified sewers (Table 2). There were no differences in 2018 before sewer modification between the control and experimental sewers in terms of larval activity (Fisher exact test, *p* = 0.23). However, in 2020, after the modification of the structure of the experimental sewers, larval activity was significantly lower than in control sewers (Fisher exact test, *p* = 0.0012). *Ae. albopictus* and *Cx. pipiens* were detected breeding in sewers in 15.1% and 21.2% of visits in 2018, respectively, while in 2020, *Ae. albopictus* and *Cx. pipiens* larvae or pupae were only detected, respectively, in 5.5 and 6.0% of visits to the non-modified sewers. In 2020, none of the visits to the modified sewers recorded mosquito larvae or pupae.

## 3. Discussion

Climate change is leading to increases in temperature, changes in rainfall patterns, and a greater likelihood of occurrence of flooding or other extreme weather events [17]. Global change also facilitates the establishment of alien species, including mosquitoes, which can provoke the transmission of pathogens [17]. Increased human mobility has led to the dispersal of pathogens such as Dengue, Zika and Chikungunya that give rise to local outbreaks whenever and wherever viraemic humans and competent vectors coexist (e.g., Dengue outbreaks in France and Spain [11]). Consequently, the implementation of measures to lessen mosquito reproduction in urban areas will have important positive consequences for public health.

Mosquito larvae were less frequently found in 2020 than in 2018, probably due to the reduced water precipitation in 2020 that may have reduced the availability of flooded sewers. Interestingly, in 2020 mosquito larvae were only observed in control sewers and no larvae were found in modified sewers. The results of this pilot experiment demonstrate how a rapid and low-cost modification of sewer structure makes sewers unsuitable places for mosquito reproduction and so precludes the need to undertake specific monitoring or biocide treatments. Similar projects have successfully reduced the presence of *Ae. aegypti* in Brazil [18]. However, this approach has some limitations, because it can only be used in sand sewers and is not applicable for use in sewers with siphonic structures. Siphonic sewers have a different function, i.e., to prevent the seepage of bad odours, and are usually located in the older districts of cities where sewer systems are less modern and can still cause this type of problem. Sand sewer structure was aimed to prevent sand from entering the sewage system, thus avoiding problems of blockages and clogging. It might seem that modification of a large number of sand sewers could be problematic, but the structural changes carried out in the sewer structure favouring the quick circulation of water has meant that incorporating sand into the system is no longer a problem, favouring the implementation of these interventions. In the case of Barcelona, approximately 8000 sand sewers could potentially be modified, which would guarantee a reduction both in the annual cost of mosquito surveillance and control programs and in the maintenance of these structures. Overall, there would potentially be a reduction by 50 kg/year in the amount of biocides used.

The control of mosquito breeding in sand sewers using larvicides requires the regular application of products to these breeding sites, partly because rain and the cleaning of streets with water washes the biocides away. Applications have to be performed repeatedly during the mosquito breeding season every year, given that larvicides persist for less than four weeks.

Sand sewer structures aim to prevent sand from entering the sewage system and causing blockages and clogging. The modification of a large number of sand sewers may appear problematic, yet the structural changes carried out in sewers to favour the rapid circulation of water has meant that the incorporation of sand is no longer a problem, and this type of intervention is highly feasible. The economic costs of the modification of sewer structures have been estimated at 350 EUR/unit; nevertheless, despite this relatively low cost per unit, the sheer number of these structures that need to be modified does raise the overall cost. The monitoring that has taken place over the years has revealed that only a few sand sewers are used every year by mosquitoes. Hence, the sewers in which mosquito activity has been recorded in previous years can be prioritized, thereby maximizing the impact of any action if financial resources are limited. This is the approach that will be applied by the Public Health Agency in Barcelona in the coming years to reduce the nuisance caused by mosquito bites and lessen the risk of disease transmission, which are regarded as key measures for reducing the impact of global change on the city. Future studies will be necessary to quantify the impact on public health of these interventions.

## 4. Materials and Methods

The city of Barcelona is located on the Mediterranean coast of Spain and its over 1,664,182 inhabitants extend over a surface area of 101.4 km^2^ [19]. It has more than 80,000 elements of different types that convey water to the sewage system. About 15% of these elements are liable to accumulate and retain water, and so can become breeding sites for mosquitoes. This is the case of both siphonic and sand sewers. Siphonic sewers aim to prevent sewage odours, and the city only possesses a few such sewers. Sand sewers, on the other hand, are more abundant, especially in the city’s parks; they have small basins and an overflow pipe for excess water, help accumulate the sand washed down by the rain and so prevent excess sedimentation in the sewerage system. However, along with this sand, a small volume of water where mosquitoes can breed is also retained. These sites are dark, with a suitable temperature and plenty of organic matter, and are the main breeding ground for mosquitoes in public areas. As part of the mosquito surveillance and control programme, all these sewers and fountains are visited regularly (usually every four weeks), and each element of risk is inspected. Biological products with active larvicides are applied if larval activity is detected. These monitoring activities are intensified during the months in which vector activity is most intense (April–November).

### 4.1. Reduction of Sand Sewer Availability for Mosquitoes

For this study, we selected 39 sand sewers in which regular larval activity of *Ae. albopictus* and *Cx. pipiens* had been recorded before 2018. The monitoring of the sand sewers was performed between 4 April and 14 December 2018; each sewer was visited at approximately monthly intervals (range: 5–10 visits/sewer). At each visit, the presence of water was determined. Technicians sampled waters for mosquito larvae using a plastic dipper (0.5 L capacity) with a long handle (1.5 m). Sampling was carried out by collecting up to one litre of water from the surface of the sewer and, if not possible due to the low water level, larvae and/or pupae were collected with a hand collector. If larvae were present, the sewer was treated using a formulation of *Bacillus thuringiensis israelensis* and *Bacillus sphaericus* (BTI treatment) using 10 g/sewer of Vectomax FG (Valent Biosciences Corporation, Libertyville, IL, USA), and all the treatments conducted were recorded. The collected larvae and pupae were placed in plastic containers, labelled with the date of collection and the sewer of origin, and transported to the laboratory of the city’s Public Health Agency. Larval specimens were identified using morphological keys [20].

**Figure 2:**
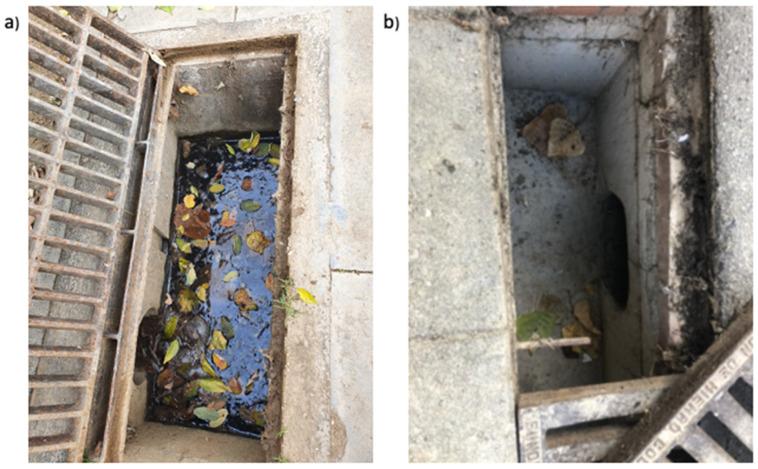
Sand sewer with accumulated water before the modification (**a**) and modified sand sewer with its bottom filled with concrete to the level of the base of the discharge pipe (**b**).

During 2019 the structure of 19 sand sewers was transformed by adding a layer of concrete that reached up to the lower level of the discharge pipe and sloped toward the pipe to prevent water accumulation (Figure 2). In 2020, the sand sewers were visited at approximately two-week intervals between 27 April and 25 November (range: 6–13 visits/sewer) to record mosquito activity. Rainfall between April and November was 988 mm in 2018 and 723.5 mm in 2020.

### 4.2. Statistical Analyses

Due to the lack of variability in some of the season/treatment combinations, it was not possible to use Generalized Linear Mixed Model (GLMM) with a binomial distributed error to analyse the data. After manipulation, all the manipulated sewers had 0 larvae in all visits, and this lack of variability means that GLMM did not reach convergence. Consequently, we calculated the percentage of the visits to each sewer with water present at the bottom of the sewer (water presence), with mosquito larvae or pupae (mosquito presence), or with BTI treatment applied (treatment). Year, sewer manipulation treatment and the two-way interaction were included as fixed factors; sewer identity was controlled as a random factor in a GLMM with normal distributed errors and identity link in JMP 9.0.1 (SAS Institute). Models were fitted by Restricted Maximum Likelihood. When the interaction between year and sewer manipulation was significant, the significance between factor levels were tested using test slices. In addition, we compared the number of sewers with and without mosquito larval activity between the control and experimental sewers in 2018 (before modification) and 2020 (after modification) using Fisher’s exact test.

## Figures and Tables

**Table 1:** Results of univariate Generalized Linear Mixed Model (GLMM) testing the effect of year (2018 vs. 2020), treatment (control vs. modified) and the two-way interaction (Year*Treatment) on the percentage of visits with water, with mosquito larvae or pupae, and with the BTI application. Sewer identity was included in the models as a random factor.

	Year		Treatment		Year*Treatment	
	F_1,37_	*p*	F_1,37_	*p*	F_1,37_	*p*
Water presence	90.49	<0.0001	29.34	<0.0001	16.68	0.0002
Mosquito presence	32.35	<0.0001	0.83	0.37	3.29	0.08
BTI application	141.89	<0.0001	7.74	0.009	5.92	0.02

**Table 2:** Mean number of sewers, mean ± s.e. (range) number of visits per sewer, visits with water, visits with mosquito larvae or pupae, and BTI application in control and modified sewers before (2018) and after (2020) the modifications.

	2018		2020	
	Control	Modified	Control	Modified
Number of sand sewers	20	19	20	19
Number of visits per sewer	8.85 ± 0.37 (5–10)	8.79 ± 0.18 (8–10)	9.15 ± 0.20 (8–10)	10.58 ± 0.40 (6–13)
Number of visits with water	6.75 ± 0.52 (4–10)	6.21 ± 0.60 (2–9)	4.45 ± 0.49 (0–8)	0.05 ± 0.05 (0–1)
Number of visits with mosquito larva or pupa	1.70 ± 0.27 (0–4)	1.95 ± 0.37 (0–5)	0.70 ± 0.19 (0–2)	0
Mean number of BTI treatments applied	4.65 ± 0.42 (3–7)	4.42 ± 0.45 (1–7)	1.75 ± 0.26 (0–4)	0
Mean number of *Ae. albopictus* larvae and pupae per visit	12.23 ± 2.90 (0–231)	3.04 ± 0.75 (0–54)	3.90 ± 2.45 (0–432)	0
Mean number of *Cx. pipiens* larvae and pupae per visit	15.90 ± 4.07 (0–421)	19.07 ± 4.21 (0–385)	3.93 ± 1.46 (0–147)	0

## Data Availability

Not applicable.
